# Receptor discordance between primary tumors and nodal metastases and correlation with the 21-gene recurrence score in early-stage, estrogen receptor-positive, node-positive breast cancer

**DOI:** 10.1007/s10549-026-08009-0

**Published:** 2026-06-22

**Authors:** Serena Zheng, Peggy Sullivan, Kumkum Vadehra, Jiyoon Kim, Alexis Levee, Rena Callahan, Mediget Teshome, Chi-Hong Tseng, Aditya Bardia, Nimmi S. Kapoor

**Affiliations:** 1 Department of Surgery, University of California Los Angeles, 10833 Le Conte Ave. 54-117 CHS, Los Angeles, CA 90095 USA; 2 Department of Pathology and Laboratory Medicine, University of California Los Angeles, Los Angeles, CA USA; 3 Department of Biostatistics, University of California Los Angeles, Los Angeles, CA USA; 4 Department of Medicine, University of California Los Angeles, Los Angeles, CA USA

**Keywords:** Node-positive breast cancer, Receptor discordance, HER2-low, Triple negative breast cancer, Oncotype Dx, ER-positive

## Abstract

**Background:**

In estrogen receptor (ER)-positive, HER2-negative breast cancer, systemic therapy decisions are often made on the biomarker status of the primary tumor, along with the Oncotype DX Recurrence Score^®^ (RS). Biomarker testing of nodal metastases is not routine, despite reported receptor discordance. We quantified ER/PR/HER2 discordance between primary tumors and axillary lymph node metastases and evaluated associations with RS and clinicopathologic features.

**Methods:**

We conducted a retrospective study of women with stage II–III, ER-positive, HER2-negative, node-positive invasive breast cancer who underwent upfront surgery and Oncotype DX testing of the primary tumor between 2016 and 2020. ER, PR, and HER2 immunohistochemistry (IHC) was performed on archived nodal metastases. Discordance in ER/PR percent positivity and HER2 IHC score between primary and lymph node metastases was scored ordinally from 0 to 2 for no, mild, or marked difference, respectively. Associations with RS, tumor and nodal burden, and treatment were analyzed using standard parametric and nonparametric tests with statistical significance set at *p* < 0.05.

**Results:**

Of 555 patients with RS testing, 91 patients met inclusion criteria and 73 had available nodal tissue. Discordance in at least one receptor was high overall, occurring in 56/73 (77%) cases. The discordance rate for ER was 15%, PR was 32%, and HER2 was 66% (58% mild, 8% marked). One case demonstrated loss of ER positivity in the node, and 14 demonstrated loss of PR positivity in the node; no primary HER2-negative tumor converted to HER2-positive (IHC 3+) disease in the node. Greater amount of HER2 discordance was associated with larger primary tumors and larger size of nodal metastatic deposits. Higher RS correlated with lower primary ER and PR expression and higher volume of nodal burden (*p* < 0.05 for each) but was not significantly associated with receptor discordance.

**Conclusions:**

Receptor discordance between primary tumors and nodal metastases is common, with most shifts occurring within the HER2-spectrum. There was a trend toward higher RS and ER or HER2-receptor discordance. HER2 discordance was also associated with larger tumor size and larger size of nodal metastasis. As strategies emerge to target HER2-low cohorts and to include ER-low/HER2-negative disease within treatment regimens for TNBC, it may become important to consider receptor testing of nodal disease in the future.

**Supplementary Information:**

The online version contains supplementary material available at 10.1007/s10549-026-08009-0.

## Introduction

Successful treatment of breast cancer relies on accurate biomarker characterization of the tumor—identifying the expression levels of estrogen receptor (ER), progesterone receptor (PR), and human epidermal growth factor receptor 2 (HER2) determine both treatment and prognosis [1]. Current national guidelines dictate that patients with breast cancers that have ER expression of 1% or higher on immunohistochemistry (IHC) [2] benefit from hormonal blockade [3]. At the same time, patients with low ER or PR expression are known to have worse overall prognosis than patients with high ER and/or PR expression, and ASCO/CAP guidelines recommend describing ER 1–10% as “low-positive” while others recommend including ER 1–10% positive and PR 1–10% positive with triple-negative breast cancer (TNBC) as seen in the inclusion criteria and definition of TNBC for the NeoPACT trial [4–7]. Meanwhile, patients with HER2-positive breast cancer, defined as IHC 3 + or confirmed gene amplification by in situ hybridization (ISH), benefit from HER2-directed treatment [8]; however, the new classification of HER2-low and HER2-ultralow expression, distinct from HER2-negative and HER2-positive disease and defined by 0+ (HER2-ultralow) and 1 + or 2+ (HER2-low) IHC staining with negative amplification on ISH, also has important treatment implications [9–11]. Results from the phase 3 DESTINY-Breast04 trial, for example, show that the HER2-targeting antibody-drug conjugate (ADC) trastuzumab deruxtecan, approved for use in metastatic HER2-positive breast cancer, provided significantly better progression-free and overall survival compared to physician’s choice of chemotherapy in patients with pretreated HER2-low metastatic breast cancer [10]. 

In addition to standard breast cancer biomarkers, the Oncotype DX Breast Recurrence Score^®^ (RS) is a 21-gene assay which provides prognostic and predictive information on the benefit of chemotherapy in ER-positive, HER2-negative breast cancer [12]. Initially, Oncotype DX was validated in the phase 3 TAILORx trial which showed that women with node-negative disease and RS of 11–25 derived little absolute benefit from adjuvant chemotherapy, with a modest benefit confined largely to younger or premenopausal patients with RS of 16-25 [13]. Oncotype DX has since been validated in node-positive breast cancer in the phase 3 RxPONDER trial which included women with 1–3 positive nodes and RS 0–25. The trial showed that chemotherapy improved outcomes for premenopausal women with 1–3 positive nodes and RS less than 25, but not for postmenopausal women, underscoring the role of RS in refining chemotherapy recommendations by menopausal status and nodal burden [14]. 

Current available data regarding adjuvant systemic therapies and survival outcomes in breast cancer have been based on biomarker profiling of the primary breast tumor only, without requirement of lymph node biomarker profiling. Yet there is ample evidence of biomarker discordance between breast primary tumors and concurrent lymph node and/or other distant metastases [15–17]. Biologically, this is thought to occur as a part of tumor evolution and immune evasion. Rates of ER and PR discordance have been reported to be between 3 and 30% and 3–32%, respectively, and rate of HER2 discordance have been reported between 0 and 14% [18]. Although previous studies have suggested the benefit of lymph node biomarker analysis, this has not been a standard practice and is not currently required for clinical trial enrollment in early-stage breast cancer.

In this study, we determine the rates of hormone receptor (ER and PR) and HER2 discordance between primary breast tumors and concurrent axillary lymph node metastasis in ER-positive breast cancer and explore associations with markers of disease burden and RS.

## Methods

### Study design and patient population

**Fig. 1:**
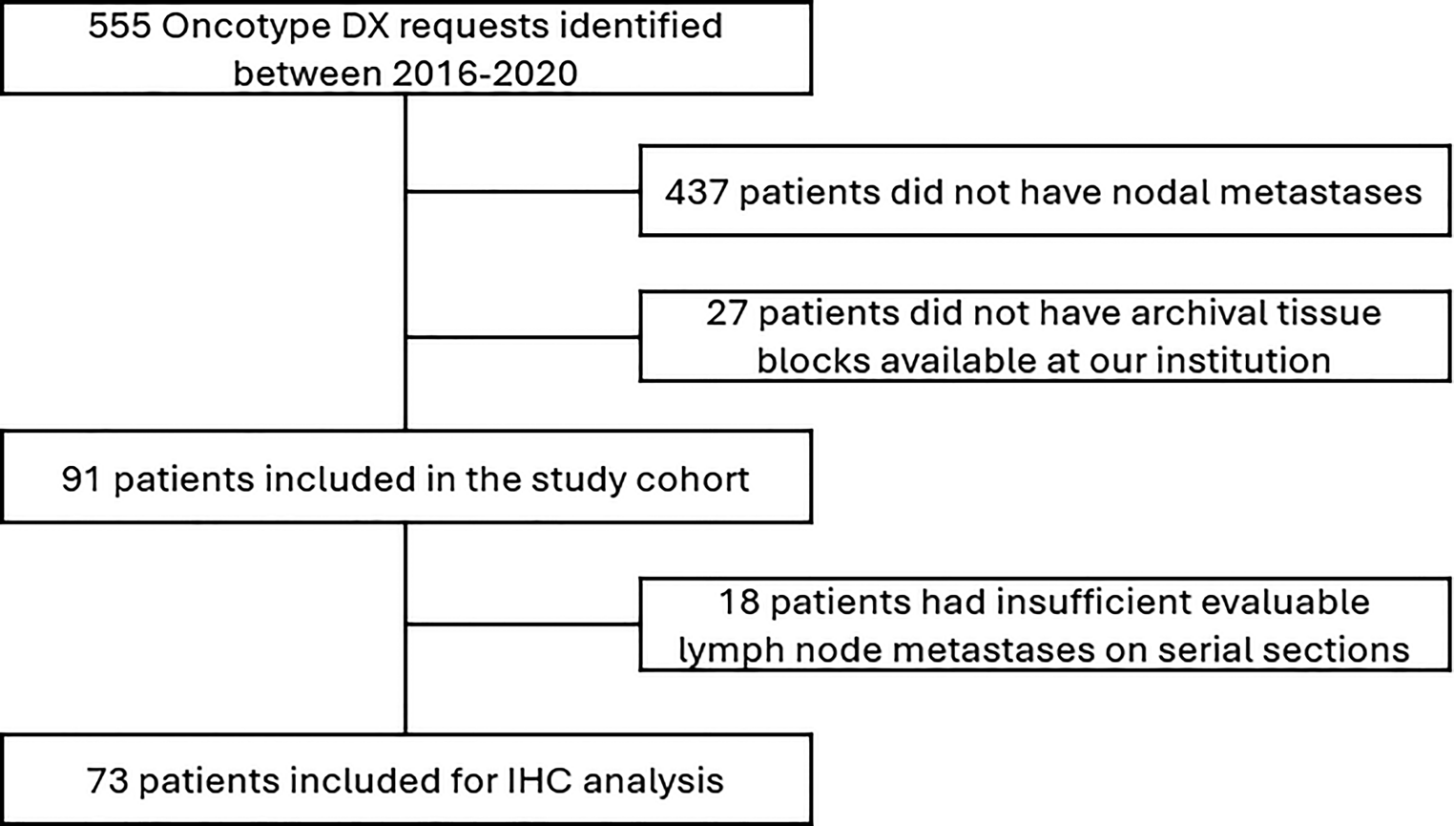
Consort diagram of patient selection for analysis. Abbreviations: IHC, immunohistochemistry; Oncotype Dx, Oncotype Dx Recurrence Score

We conducted a retrospective observational cohort study at a single academic center (University of California, Los Angeles [UCLA]). We identified female patients ≥18 years who were diagnosed with stage II-III, hormone receptor-positive, HER2-negative, node-positive invasive breast cancer who underwent upfront surgery with Oncotype DX testing of the primary breast tumor between 2016 and 2020 (Fig. 1). Patients who underwent neoadjuvant chemotherapy or were found to have Stage IV disease at time of diagnosis were excluded. Patients were excluded from nodal biomarker analysis if they had minimal lymph node disease burden (i.e. isolated tumor cells) or insufficient remaining nodal metastatic tissue for biomarker analysis. Additionally, patients were excluded if they had only non-invasive disease (e.g., ductal carcinoma in situ [DCIS]), any prior treatment (e.g., chemotherapy or immunotherapy) for breast cancer, concurrent estrogen receptor (ER)-negative primary tumors, or HER2-positive primary tumors by American Society of Clinical Oncology/College of American Pathologists (ASC/CAP) criteria (e.g., IHC score of 3 + or ISH-amplified).

### Clinical assessment

Clinicopathologic variables were abstracted from the medical record via manual chart review. Variables collected included demographics, menopausal status, tumor characteristics, receptor status of the primary tumor and nodal disease, nodal burden, type of breast surgery and axillary procedure, treatment variables, and locoregional recurrence, distant recurrence, and survival. Oncotype DX RS (primary tumor, Genomic Health [Exact Sciences]) was also abstracted from the electronic medical record. Patients were stratified based on RS ≤ 15 or ≥ 16, similar to the TAILORx trial instead of higher cut-offs as seen in RxPONDER due to the lower median RS of 15 seen in this study.

### Specimen processing

Pre-treatment diagnostic biopsies and surgical resection specimens were processed through standard diagnostic pathology workflows and access to remnant pathologic material was IRB exempt. Surgical pathology cases were reviewed by a single specialized pathologist (PS) who selected appropriate formalin-fixed paraffin-embedded (FFPE) tissue blocks from which standard H&E stained and serial unstained histologic sections for downstream analysis were generated by the UCLA Translational Pathology Core (TPCL) Facility.

### Immunohistochemical staining of human samples

Receptor status of the primary tumors was obtained by chart review of pathology reports of biopsy and surgical resection specimens. Lymph node sections from a single archived lymph node metastasis were subsequently stained for receptor comparisons. The Translational Pathology Core Laboratory sectioned select formalin-fixed paraffin-embedded (FFPE) blocks and performed hematoxylin and eosin (H&E) and IHC staining on serial sections. IHC staining was performed using the Leica Bond RX processor with the Bond Polymer Refine Detection kit (Leica Biosystems, Cat. DS9800; Protocol F). Briefly, following the instrument’s factory based “Bake & Dewax” protocol, heat-induced epitope retrieval was performed with BOND ER1 buffer (Leica Biosystems, Cat. AR9961) at 100 °C for 20 min (min) and then incubated with Peroxide Block for 5 min. Primary antibodies were applied for 60 min at the following dilutions: HER2/ERBB2 (Cell Signaling Technology #4290, D8F12 XP, 1:200), ER (Biocare Medical ACA301, 1:100), and PR (Biocare Medical ACA302A, clone SP2, 1:50). Detection used the kit’s post-primary and HRP polymer reagents (10 min and 8 min, respectively), with buffered rinses between steps per manufacturer instructions. Chromogen development used DAB Refine (10 min) followed by hematoxylin counterstain (10 min). Slides were dehydrated through graded alcohols, cleared with histoclear, and mounted with Permount. All slides were examined and annotated by a single, specialized pathologist (PS).

Discordance between paired sites (primary tumor vs. lymph node metastasis) was scored ordinally. ER and PR percent positivity discordance between primary and nodal staining was scored “0” if the difference in percent positivity between the primary tumor and the nodal metastasis was < 25%, “1” (considered mild) for a difference between 25 and 50%, and “2” (considered marked) if there was > 50% difference. HER2 intensity discordance was scored “0” for no differences in IHC stains, “1” (mild) for 1-point difference (e.g., 1 + vs. 2+), and “2” (marked) for > 1-point difference.

### Statistical analysis

Summary statistics were generated for patients’ demographic and clinical variables to characterize the study population. Chi-square or Fisher’s exact test was used to compare categorical variables, and t-test or Wilcoxon’s test was used to compare continuous variables. Associations between clinical predictors (tumor size, node size, Oncotype Score, and number of positive lymph nodes) and biomarker expression levels (ER, PR, and HER2) were evaluated using continuous, ordinal (levels 0, 1, and 2) and binary (levels 1 and 2 vs. 0) outcome frameworks. For continuous and ordinal outcomes, analysis of variance (ANOVA) and ordinal logistic regression were performed. For binary outcomes, two-sample t-tests and binary logistic regression were used. Statistical significance was defined as *p* < 0.05. All analyses were conducted using R (version 4.3.3).

## Results

### Patient characteristics

Of 555 patients with RS testing during the study period, 91 patients had node-positive disease and met study criteria and were included for analysis. Patient characteristics are summarized in Table 1. Median patient age at diagnosis was 56 years (range 33–80 years) and 62% were postmenopausal. Germline testing was performed on 45% of patients, of whom 4.4% carried a pathogenic mutation.

### Histopathological characteristics

The median primary tumor size was 2.1 cm (range, 0.6–9.8 cm). 25% of patients had multifocal tumors. Histologically, 81% of patients had invasive ductal carcinoma (IDC), 15% had invasive lobular carcinoma (ILC), and 3% had mixed/other histology.

In the primary tumor, ER percent positivity was high overall: 71% of tumors had > 90% ER-positive cells and 29% had 10–90%; no cases had ≤ 10% positivity. In terms of PR percent positivity, 22% of tumors had > 90% positivity, 59% had 10–90% positivity, and 19% had ≤ 10% positivity. Overall, 97% of tumors were ER-positive/PR-positive, and 3% of tumors were ER-positive/PR-negative. HER2 IHC was 0 in 14%, 1 + in 56%, and 2 + in 30%. In line with study inclusion criteria, no patients had a primary breast tumor HER2 IHC score of 3+.

With regards to pathologic nodal status, 80% of patients had one positive node, 19% had two positive nodes, and 1% had three or more positive nodes. Nodal metastases volume measurement showed that 36% of cases included had micrometastases (nodal metastasis ≤ 2 mm) and 64% had macrometastases (nodal metastasis > 2 mm in size). No cases had isolated tumor cells as the only type of nodal metastasis.

### Oncotype Dx recurrence score

The median RS was 15 (range 1–42), with 50 patients in the RS ≤ 15 group and 41 in the RS ≥ 16 group. Groups were similar across most clinicopathologic features—including age at diagnosis, menopausal status, histology, tumor size, and grade (Table 1). As expected, adjuvant chemotherapy use was higher in the RS ≥ 16 group (44%) than in the RS ≤ 15 group (14%, *P* = 0.006). Tumors in the RS ≥ 16 group were characterized by lower hormone-receptor expression: PR ≤ 10% occurred in 34% in the RS ≥ 16 group vs. 6% in RS ≤ 15 (*P* = 0.003), and ER 10–90% in 39% vs. 20% (*P* = 0.046). The RS ≥ 16 group also showed greater axillary burden, with > 1 positive node in 29% vs. 12% (*P* = 0.047) (Table 1).

### Treatment and follow-up

57% of patients underwent partial mastectomy, and 43% underwent total mastectomy. 96% of patients had a sentinel lymph node biopsy, and 4% had an axillary lymph node dissection. 82% of patients received radiation, 15% did not receive radiation, and 2% had unknown radiation treatment. Adjuvant chemotherapy was administered to 27% of patients overall— including 14% of patients with RS < 16, 38% with RS 16–25, and 72% with RS > 25. Most (91%) patients received adjuvant endocrine therapy for a median duration of 4 years (range 0–9). Median follow-up time was 5.0 years (0-9.5 years). Locoregional recurrences occurred in 2% of patients and distant recurrences in 8%. Deaths were recorded in 4 out of 91 patients (4%). The low number of events in the follow-up period precluded identification of associations between receptor discordance and outcomes.

### Receptor discordance

**Fig. 2:**
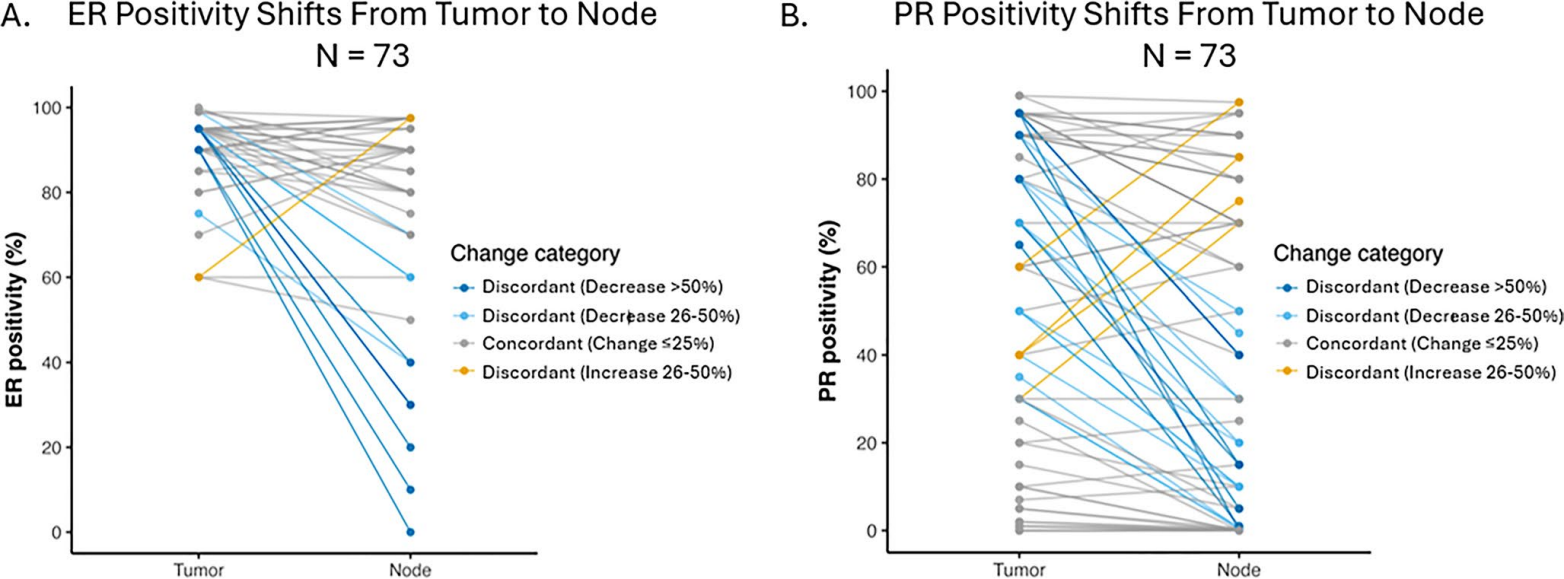
Hormone receptor shifts between primary tumor and lymph node (**A**) Comparison of ER positivity in primary breast tumors and matched nodal metastases. (**B**) Comparison of PR positivity in primary breast tumors and matched nodal metastases. Each line represents an individual patient. Discordance was categorized according to absolute change in receptor positivity: score 0, change ≤ 25%; score 1, change > 25% to 50%; and score 2, change > 50%. In one case in panel B, nodal PR positivity was reported as 5–10%; for graphical display, this value was coded as 10%. Abbreviations: ER, Estrogen Receptor; PR, Progesterone Receptor

Out of 73 patients with archived nodal metastasis tissue available for IHC analysis, 56 (77%) had discordance in at least one receptor (ER, PR, or HER2) (Supplemental File). For ER and PR, discordance primarily reflected decreases in percent positivity (with rare increases), whereas HER2 showed both upward and downward shifts in IHC category (Table 2; Fig. 2A and B). More discordance overall was observed in the HER2 biomarker compared to ER or PR (Table 2).

#### ER discordance

All primary tumors were ER-positive, in line with the inclusion criteria, and only one had concurrent nodal metastases with ER-negative staining (IHC score 0) (Supplemental File). The majority of cases demonstrated concordant ER expression between the primary tumor and nodal metastasis (score 0, 85%) (Fig. 2A). Discordance was observed in 11/73 (15%) patients: 5 (45%) with a mild discordance score of 1, and 6 (55%) with a marked discordance score of 2, including one case with ER-low staining in the node (10%) but ER-positive staining in the primary (90% staining). No significant associations were observed between tumor sizes (*p* = 0.72), number of positive nodes (*p* = 0.92), or size of nodal metastases (*p* = 0.65). The RS trended higher with higher discordance scores; the mean RS was 15.5 in concordant cases, 16.0 in mild discordant cases (score 1), and 22.0 in marked discordant cases (score 2) (*p* = 0.087).

#### PR discordance

Most (95%) patients had PR-positive tumors. Notably, 14/73 (19%) patients who had PR-positive primary tumors had PR-negative nodal metastases (IHC PR positivity < 1%) and an additional 4 (5%) patients had PR positivity 1–10% nodal metastasis (Supplemental File). Similar to ER expression, the majority (50/73, 68%) of cases demonstrated concordant PR expression between the primary tumor and nodal metastasis (score 0) (Supplemental File). Mild discordance (score 1) was observed in 16/73 (22%) patients, and marked discordance (score 2) was observed 7/73 (10%) patients (Fig. 2B). In one case, lymph node PR positivity was reported as 5–10%; for graphical display in Fig. 2B, this value was coded as 10%. No significant differences were observed between PR discordance and tumor size (*p* = 0.66), number of positive nodes (*p* = 0.55), or size of nodal metastases (*p* = 0.44). RS values did not significantly differ across discordance groups (*p* = 0.73).

#### HER2 discordance

**Fig. 3:**
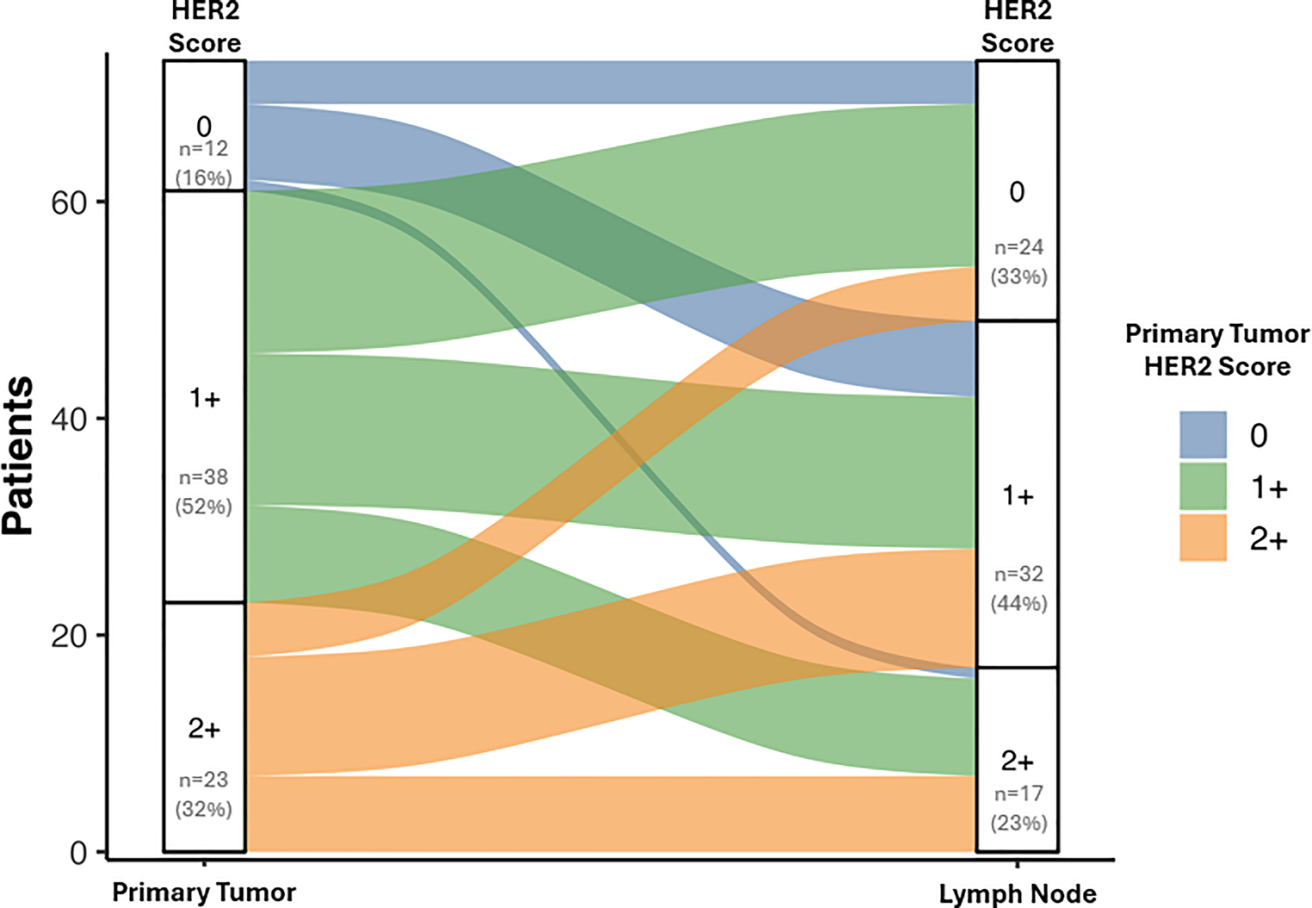
HER2 shifts between primary tumor and lymph node. Comparison of HER2 immunohistochemistry (IHC) scores in primary breast tumors and matched nodal metastases. Each line represents an individual patient. HER2 discordance was categorized by ordinal IHC shift: score 0, no change; score 1, change of 1 category; and score 2, change of more than 1 category. In one case, nodal HER2 was reported as a range (0–1+); for purposes of graphical representation, this value was classified as HER2 1+. Abbreviations: HER2, Human Epidermal Growth Factor Receptor 2

HER2 IHC discordance occurred in 48/73 (65.8%) patients (Table 2). Of these 48 patients, 17 (35.4%) had increased HER2 IHC scores in the lymph node compared to the primary tumor including 8 cases with a shift from HER2-negative (IHC 0) in the primary tumor to HER2-low (IHC 1 + or 2+) in the nodal deposit (Fig. 3). In one case, nodal HER2 was reported as a range (0–1+); for purposes of graphical representation in Fig. 3, this value was classified as HER2 1+. No cases had HER2-negative primary tumors with concurrent HER2-positive (IHC 3 + staining) nodal metastases (Table 2). Most (42/48, 87.5%) discordant cases classified as mild discordance (score 1) and 6/48 (12.5%) cases as marked (score 2). Tumor size in concordant cases averaged 1.9 cm, compared with 2.9 cm in mildly discordant cases and 1.8 cm in markedly discordant cases (ANOVA, *p* = 0.037). Pairwise comparisons showed larger tumors in mildly discordant (score 1) vs. concordant (score 0) cases (*p* = 0.007) as well as compared to the markedly discordant group (score 2) (*p* = 0.028). Additionally, mean nodal metastatic deposit size was smallest in concordant cases (3.1 mm), and larger in both mildly discordant (6.1 mm) and markedly discordant cases (5.9 mm), pairwise comparison of score 0 vs. score 1 *p* = 0.008. RS also trended higher in discordant groups; mean RS was 14.6 in concordant cases, 16.5 in mild discordant cases, and 19.5 in marked discordant cases (*p* = 0.26). Number of positive nodes were similar between groups.

In logistic regression, a larger tumor size was significantly associated with higher odds of HER2 IHC discordance (OR = 1.668, 95% CI: 1.015–2.742, *p* = 0.044). Size of nodal metastatic deposit was also associated with HER2 IHC discordance, showing significance in ordinal logistic regression (OR = 1.108, 95% CI: 1.008–1.218, *p* = 0.034).

## Discussion

In this single-center cohort of early stage, hormone receptor-positive, HER2-negative, node-positive breast cancer, biomarker discordance between the primary tumor and the nodal metastasis was evaluated and compared to the primary tumor’s Oncotype DX RS. Among the 73 cases with archived nodal tissue available for review, discordance was common: 77% demonstrated discordance in at least one receptor. This was largely driven by HER2 variability within the HER2-negative spectrum, with 66% of cases exhibiting any HER2 discordance (58% mild; 8% marked). Despite high rates of discordance, no case converted from HER2-negative primary tumor to HER2-positive metastatic lymph node, but 11% of cases did shift from HER2-negative to HER2-low, including 1 case that was HER2 0–1+. Notably, our HER2 discordance rate is higher than many previously reported studies because discordance in our series is defined as any shift in HER2 staining, including within the HER2-negative spectrum (IHC 0 vs. 1 + vs. 2+), rather than restricting discordance to changes that cross the traditional HER2-positive threshold (IHC 3 + or ISH-amplified). As a result, our findings primarily capture within-category variability that may reflect true biologic heterogeneity, sampling differences, as well as the known analytic sensitivity limits of semiquantitative HER2 IHC scoring.

Tumor size varied across HER2 discordance categories (ANOVA *p* = 0.037), with the largest tumors in the mildly discordant (score 1) group compared to the concordant and markedly discordant groups (*p* = 0.007). Larger nodal metastatic deposit size tracked with increasing levels of HER2 discordance on the ordinal scale (OR 1.108, 95% CI 1.008–1.218; *p* = 0.034). The association between HER2 discordance and both primary tumor size and nodal metastatic deposit size suggests that higher burden of disease may be comprised of more heterogeneous disease, thereby increasing the likelihood that a single sampled region will not fully represent the axillary phenotype. Practically, these findings may support a targeted approach to nodal re-testing that prioritizes cases with disproportionately bulky nodal disease or borderline primary HER2 results.

The lack of true HER2 positivity seen the nodal analysis of this study is similar to one conducted by Janeva et al., who also found, in a study of 94 breast cancer patients, no HER2 status changes from negative in the primary tumor to positive in the lymph node metastasis [18]. However, the high rate of HER2 variability (IHC 0/1+/2+) between sites in our study, coupled with the observed associations with tumor and nodal burden, highlights a phenotype that may become relevant if ongoing trials demonstrate benefit for HER2-low and ultralow–directed strategies, especially in early-stage disease.

The emergence of HER2-low (IHC 0+/1+/2+, ISH-negative) disease as a distinct entity and therapeutically actionable subset has reshaped how HER2 is considered in clinical practice and in trials [19, 20]. In DESTINY-Breast04, patients with metastatic, pretreated HER2-low breast cancer experienced significantly improved progression-free and overall survival with trastuzumab deruxtecan compared with physician’s choice chemotherapy, establishing HER2-low as a distinct treatment subset within the historical HER2-negative category [10]. Similarly, DESTINY-Breast06 demonstrated benefit of trastuzumab deruxtecan in HER2 ultralow (0+) metastatic disease further supporting the clinical relevance of detecting any amount of HER2-IHC staining [21]. These results support more granular HER2 assessment and reporting as even small shifts in IHC category (0 vs. 0 + or 1 + vs. 2+) can alter eligibility for HER2-targeted ADCs in advanced disease [21–23]. Clinical trials of trastuzumab deruxtecan are ongoing in the early-stage setting, which may influence the need to report HER2-low and ultralow status in this patient population.

Utilizing an ordinal scoring system, rates of discordance in ER and PR were less common than seen with HER2, occurring in 15% of cases for ER and 32% of cases for PR 32%. However, one case converted from ER-positive in the primary tumor to ER-negative in the lymph node, a second case converted to ER-low; both of these cases (Supplemental file) were also consistent with a re-emerging definition of TNBC with both ER and PR in these two cases being 10% or less. Moreover, while RS did not significantly associate with HER2 or PR discordance, there was a non-significant trend with higher RS scores and ER discordance.

A large population-based study out of Sweden, where ER < 10% is considered negative, showed that of 5655 tumors, ER-low, HER2-negative breast cancer, when treated the same as TNBC tumors, had similar prognosis to TNBC with no difference in pathologic complete response (pCR) or overall survival [7]. While additional smaller studies have confirmed these findings and have shown efficacy of pembrolizumab for ER-low TNBC [24, 25], the NeoPACT phase 2 clinical trial of pembrolizumab with an anthracycline-free regimen of neoadjuvant chemotherapy purposely included TNBC to allow for ER and PR of 10% or less [5]. Similar to the HER2 landscape, all these studies are currently only evaluating primary tumor receptors; nodal testing is not considered. Larger studies with extended follow-up are needed to assess whether ER nodal discordance is associated with differences in survival outcomes and thereby could benefit from TNBC treatment strategies.

Specifically, while immunotherapy regimens in ER+ breast cancer are awaiting long-term survival data and are not yet standard of care, there may be subset of ER+ disease with nodal ER-negative/ER-low pathology that could presumably derive more benefit from immunotherapy regimens. In KEYNOTE-756 for example, patients with high-risk ER+HER2- disease were randomized to immunotherapy (pembrolizumab) or placebo and neoadjuvant chemotherapy [26]. Immunotherapy significantly improved pCR rates, and patients with ER-low disease (ER < 10%) had higher pCR rates than those with higher ER-positivity.

Multiple series have demonstrated discordance between primary tumors and synchronous nodal metastases across ER, PR, and HER2, with reported any-marker discordance ranging from approximately 20% to 50% [27, 28]. For example, in one study of 101 patients with breast cancer, ER status of the primary breast lesion was compared to that in lymph node metastases, and discordant expression was found in 21% of patients [29]. In a second study of 385 patients, rate of discordance of either ER, PR, or HER2 was 46.9%, with clinically significant changes in 23.1% of cases [30]. Other studies also describe internodal heterogeneity—variation in HER2 status among different positive nodes from the same patient—highlighting the limits of single-sample assessment [31–33]. Such differences likely reflect a combination of intra-tumoral heterogeneity in the primary tumor and selection pressures within the tumor microenvironment during lymphatic spread, yielding metastatic deposits that are phenotypically non-identical to the index lesion.

Oncotype DX is a 21-gene quantitative RT-PCR assay that generates a RS that is prognostic for distant recurrence risk and predictive of chemotherapy benefit in hormone positive, HER2-negative disease. In the TAILORx which included patients with node negative disease, most women with RS 11–25 derived minimal average benefit from adjuvant chemotherapy, with an important nuance—younger/premenopausal patients (or those ≤ 50 years) with RS 16–25 showed modest benefit from chemotherapy, which may partly reflect ovarian function suppression from chemotherapy-induced premature menopause rather than direct chemosensitivity [13]. Clinical utility of Oncotype DX was extended to node-positive disease (1–3 nodes) in the RxPONDER trial, which demonstrated that premenopausal patients with RS 0–25 benefit from chemotherapy, whereas postmenopausal patients with the same RS range generally do not [14]. Together, these trials support the use of Oncotype DX in current clinical practice for patients with hormone receptor-positive disease to guide the use of adjuvant chemotherapy.

In our study, RS inversely correlated with ER and PR expression and positively with proliferation (e.g., Ki-67). This suggests that tumors with lower ER/PR percentages or higher grade/proliferation may have higher risk of recurrence as reflected by the higher RS score. Consistent with current trial-informed practice, chemotherapy use increased with higher RS (14% in RS < 16 vs. 44% in RS ≥ 16). Higher RS aligned with lower hormone-receptor expression at the primary site and with greater axillary burden, echoing the biological link between RS and adverse clinicopathologic features. Notably, RS did not meaningfully associate with hormone receptor or HER2 discordance.

Taken together, our findings support prior studies which compare Oncotype Dx on paired primary and nodal specimens. Boolbol et al. evaluated 84 paired primary tumors and synchronous nodal macrometastases using Oncotype DX and found modest correlation of continuous RS values (Pearson *r* = 0.69) with 63% concordance in RS risk group, yet categorical ER and HER2 status by RT-PCR was 100% concordant between sites and PR was concordant in 77%; they concluded that RS testing for ER-positive/HER2-negative disease should continue to be based on primary tumor tissue [34]. Our data complement these results at the protein level: despite frequent semi-quantitative shifts in HER2 and PR IHC scores between primary tumor and node, RS did not meaningfully differ by discordance category, and we observed no conversion to true HER2-positive or hormone receptor–negative disease in the lymph node. Together, these observations support the concept that RS and IHC-defined receptor discordance capture related but distinct dimensions of tumor biology—RS reflecting the overall transcriptional program and proliferation of the primary lesion, and discordance reflecting regional heterogeneity during nodal colonization—without current evidence that nodal discordance alone should prompt deviation from primary-tumor–based RS guidance.

Nodal biomarker testing may add value when primary results are borderline or equivocal, or when bulky nodal disease raises concern for phenotypic drift—particularly relevant if HER2-low–directed strategies expand into early-stage care. Prospective studies integrating RS, quantitative IHC, and multi-node sampling with outcomes will clarify when discordance warrants treatment modification beyond RS-based recommendations.

This study has several limitations. It is a retrospective, single-center analysis with a modest sample size—particularly for the 73 patients with available nodal tissue—which may introduce selection bias and limits power for multivariable analyses. Our cohort was enriched for low-to-intermediate RS values and included few patients at the extremes of genomic risk or with higher axillary burden (≥ 3 positive nodes), so generalizability to those groups is uncertain. Clinical events were rare over a median 5-year follow-up, precluding meaningful assessment of how discordance or HER2 variability affects prognosis or chemotherapy benefit. Additionally, receptor discordance was defined using ordinal categories for ER/PR percent positivity and HER2 IHC score rather than continuous or centrally quantified measures, and only one positive node per patient was evaluated; thus, smaller expression changes and internodal heterogeneity may be underestimated. Moreover, paired-site comparisons may incorporate both biologic and analytic variation. Receptor status of the primary tumor was abstracted from clinical pathology reports, whereas nodal biomarkers were assessed on archived tissue using a single staining platform and centralized pathologist review. While this contextual difference in testing is unlikely to explain large category shifts (e.g., emergence of IHC 3+), it may contribute to minor shifts within the HER2-negative spectrum (0/0+/1+/2+), where scoring is inherently semiquantitative. Finally, we restricted inclusion to ER-positive, HER2-negative tumors treated with upfront surgery between 2016 and 2020, so these findings may not extrapolate to patients with HER2-positive or triple-negative disease or those treated with neoadjuvant therapy.

## Conclusion

In this retrospective cohort study of patients with ER-positive, HER2-negative breast cancer with lymph node metastasis, we observed that 77% of patients had at least one type of receptor discordance between the primary breast tumor and their associated lymph node metastasis. There was a nonsignificant trend toward higher RS and an observed ER or HER2-receptor discordance. HER2 discordance was also associated with larger tumor size and larger size of nodal metastasis. While current treatment paradigms suggest no clinically actionable changes were identified with the level of discordance seen in this series, as strategies emerge to target HER2-low/ultralow cohorts and to include ER-low/HER2-negative disease within treatment regimens for TNBC, it may become important to consider receptor testing of nodal disease in the future.

**Table 1:** Clinicopathologic characteristics of 91 patients and Oncotype Dx Recurrence Score

		Oncotype DX Recurrence Score		
	**All (** ***N*** ** = 91)**	**< 16 (** ***N*** ** = 50)**	**≥ 16 (** ***N*** ** = 41)**	*p*-value
**Age**				
Age, median (range)	56 (33–80)	57 (33–78)	55 (37–80)	0.47
Age < 50 n, (%)	27 (30)	12 (24)	15 (37)	0.38
Age 50–65 n, (%)	44 (48)	27 (54)	17 (41)	
Age > 65 n, (%)	20 (22)	11 (22)	9 (22)	
**Race**,** n (%)**				0.78
White	52 (57)	27 (54)	25 (61)	
Non-White	18 (20)	11 (22)	7 (17)	
Unknown or Chose Not to Answer	21 (23)	12 (24)	9 (22)	
**Ethnicity**,** n (%)**				0.74
Not Hispanic or Latino	67 (74)	37 (74)	30 (73)	
Hispanic Or Latino	3 (3)	1 (2)	2 (5)	
Unknown or Chose Not to Answer	21 (23)	12 (24)	9 (22)	
**Menopausal Status**,** n (%)**				0.59
Premenopausal (incl. Perimenopausal)	35 (38)	18 (36)	17 (41)	
Postmenopausal (or Age > 59)	56 (62)	32 (64)	24 (59)	
**Genetic Mutation**				
Yes	2 (2)	0 (0)	2 (5)	0.26
No	39 (43)	23 (46)	16 (39)	
Not Tested	50 (55)	27 (54)	23 (56)	
**Tumor Subtype**,** n (%)**				0.13
Invasive Ductal Carcinoma	74 (81)	43 (86)	31 (76)	
Invasive Lobular Carcinoma	14 (15)	7 (14)	7 (17)	
Mixed/Other	3 (3)	0 (0)	3 (7)	
**Grade**,** n (%)**				0.59
Low	27 (30)	15 (30)	12 (29)	
Intermediate	54 (59)	31 (62)	23 (56)	
High	10 (11)	4 (8)	6 (15)	
**Breast Tumor Receptors**				0.088
ER + PR+HER2-	88 (97)	50 (100)	38 (93)	
ER + PR-HER2-	3 (3)	0 (0)	3 (7)	
**ER Positivity (%)**				**0.046**
≤ 10%	0 (0)	0 (0)	0 (0)	
10%-90%	26 (29)	10 (20)	16 (39)	
> 90%	65 (71)	40 (80)	25 (61)	
**PR Positivity (%)**				**0.0026**
≤ 10%	17 (19)	3 (6)	14 (34)	
10%-90%	54 (59)	35 (70)	19 (46)	
> 90%	20 (22)	12 (24)	8 (20)	
**HER2 IHC**				0.37
0	13 (14)	9 (18)	4 (10)	
1+	51 (56)	25 (50)	26 (63)	
2+	27 (30)	16 (32)	11 (27)	
3+	0 (0)	0 (0)	0 (0)	
**Ki-67**				0.71
≤ 10%	54 (59)	31 (62)	23 (56)	
11–20%	17 (19)	8 (16)	9 (22)	
> 20	16 (18)	8 (16)	8 (20)	
Unknown	4 (4)	3 (6)	1 (2)	
**Tumor Size**				
Median (range)	2.1 (0.6–9.8)	2 (0.6–9.8)	2.4 (0.7-9.0)	0.425
≤ 1 cm	7 (8)	4 (8)	3 (7)	0.25
(1–2] cm	37 (41)	23 (46)	14 (34)	
(2–5] cm	39 (43)	17 (34)	22 (54)	
> 5 cm	8 (9)	6 (12)	2 (5)	
**Multifocal**,** n (%)**				
Yes	23 (25)	13 (26)	10 (24)	0.86
No	68 (75)	37 (74)	31 (76)	
**Number of Positive Nodes**				
1	73 (80)	44 (88)	29 (70)	**0.047**
2	17 (19)	5 (10)	12 (30)	
3	0 (0)	0 (0)	0 (0)	
> 3	1 (1)	1 (2)	0 (0)	
**Largest Lymph Node Metastasis**				0.09
≤ 2 mm	33 (36)	22 (44)	11 (27)	
> 2 mm	58 (64)	28 (56)	30 (73)	
**Oncotype DX Recurrence Score**				
Median (range)	15 (1–42)	12 (1–15)	22 (17–42)	
**Breast Surgery and Radiation (Local Therapy)**				0.77
Partial Mastectomy Alone	2 (2)	2 (4)	0 (0)	
Partial Mastectomy and Radiation	49 (54)	26 (52)	23 (56)	
Mastectomy Alone	12 (13)	7 (14)	5 (12)	
Mastectomy and Radiation	26 (29)	14 (28)	12 (29)	
Unknown	2 (2)	1 (2)	1 (2)	
**Axillary Surgery**				0.84
Sentinel Lymph Node Biopsy	87 (96)	48 (96)	39 (95)	
Axillary Lymph Node Dissection	4 (4)	2 (4)	2 (5)	
**Chemotherapy Received**				**0.0058**
Yes	25 (27)	7 (14)	18 (44)	
No	64 (70)	42 (84)	22 (54)	
Unknown	2 (2)	1 (2)	1 (2)	
**Ovarian Function Suppression Received**				0.81
Yes	15 (17)	8 (16)	7 (17)	
No	74 (81)	42 (84)	32 (78)	
Unknown	2 (2)	0 (0)	2 (5)	
**Endocrine Therapy Received**				0.26
Yes	83 (91)	47 (94)	36 (88)	
No	5 (5)	1 (2)	4 (10)	
Unknown	3 (3)	2 (4)	1 (2)	
**Duration of Endocrine Therapy**				0.145
Median years (range)	4 (0–9)	4 (0–8)	4 (0–9)	
< 3 years	18 (20)	11 (22)	7 (17)	
3–5 years	50 (55)	26 (52)	24 (59)	
> 5 years	15 (16)	11 (22)	4 (10)	
No Endocrine Therapy or Unknown	8 (9)	2 (4)	6 (14)	
**Follow Up**				
Median Number of Months (range)	60 (0-113)	61 (1-113)	57 (0-113)	0.449
**Locoregional Recurrence**				0.5
Yes	2 (2)	2 (4)	0 (0)	
No	89 (98)	48 (96)	41 (100)	
**Distant Recurrence**				0.5
Yes	7 (8)	3 (6)	4 (10)	
No	84 (92)	47 (94)	37 (90)	
**Death**				0.84
Yes	4 (4)	2 (4)	2 (5)	
No	87 (96)	48 (96)	39 (95)	

**Table 2:** Breakdown of biomarker status between primary breast tumors and lymph nodes

	Category	Primary Breast Tumor	Lymph Node	Number of Patients, n (%) *N* = 73
HER2	Non-discordant HER2	HER2 (0)	HER2 (0)	4 (5)
		HER2 (1+)	HER2 (1+)	14 (19)
		HER2 (2+)	HER2 (2+)	7 (10)
		HER2 (3+)	HER2 (3+)	0 (0)
	Discordant HER2	HER2 (0,1+,2+)	HER2 (3+)	0 (0)
		HER2 (0)	HER2 (1+)	7 (10)
		HER2 (0)	HER2 (2+)	1 (1)
		HER2 (1+)	HER2 (0)	15 (21)
		HER2 (1+)	HER2 (2+)	9 (12)
		HER2 (2+)	HER2 (0)	5 (7)
		HER2 (2+)	HER2 (1+)	11 (15)
ER	Non-discordant ER	ER-	ER-	0 (0)
		ER+	ER+	62 (85)
	Discordant ER	ER+	ER positivity decrease > 25%	4 (5)
		ER+	ER positivity decrease > 50%	6 (8)
		ER+	ER positivity increase > 25%	1 (1)
		ER+	ER positivity increase > 50%	0 (0)
		ER-	ER+	0 (0)
PR	Non-discordant PR	PR-	PR-	4 (6)
		PR+	PR+	46 (63)
	Discordant PR	PR+	PR positivity decrease > 25%	12 (17)
		PR+	PR positivity decrease > 50%	7 (10)
		PR+	PR positivity increase > 25%	4 (5)
		PR+	PR positivity increase > 50%	0 (0)
		PR-	PR+	0 (0)

## Supplementary Information

Below is the link to the electronic supplementary material.

Supplementary Material 1

## Data Availability

All data for this study have been provided in the tables within the manuscript.

## References

[1] Królewska-Daszczyńska P, Englisz A, Morawiec ML, Miśkiewicz J, Gołębski M, Mielczarek-Palacz A (2025) The assessment of breast cancer biomarkers in diagnosis, prognosis and treatment monitoring: integrated analysis. J Cancer Res Clin Oncol 151(8):233. 10.1007/S00432-025-06271-1 40844552 10.1007/s00432-025-06271-1 PMC12373631

[2] Karaali C, Emiroğlu M, Değirmenci M, Keser M, Salimoğlu S, Talu CK (2023) The Clinical and Pathological Characteristics That Differentiate Cases With Low Estrogen Receptor Expression From Triple-Negative Breast Cancer. Eur J Breast Health 20(1):19. 10.4274/EJBH.GALENOS.2023.2023-6-3 38187108 10.4274/ejbh.galenos.2023.2023-6-3 PMC10765462

[3] Burciu OM, Merce AG, Cerbu S et al (2025) Current Endocrine Therapy in Hormone-Receptor-Positive Breast Cancer: From Tumor Biology to the Rationale for Therapeutic Tunning. Med (B Aires) 61(7):1280. 10.3390/MEDICINA61071280 10.3390/medicina61071280 PMC12299218 40731911

[4] Dowsett M, Allred C, Knox J et al (2008) Relationship Between Quantitative Estrogen and Progesterone Receptor Expression and Human Epidermal Growth Factor Receptor 2 (HER-2) Status With Recurrence in the Arimidex, Tamoxifen, Alone or in Combination Trial. J Clin Oncol 26(7):1059–1065. 10.1200/JCO.2007.12.9437 18227529 10.1200/JCO.2007.12.9437

[5] Sharma P, Stecklein SR, Yoder R et al (2024) Clinical and Biomarker Findings of Neoadjuvant Pembrolizumab and Carboplatin Plus Docetaxel in Triple-Negative Breast Cancer: NeoPACT Phase 2 Clinical Trial. JAMA Oncol 10(2):227–235. 10.1001/JAMAONCOL.2023.5033 37991778 10.1001/jamaoncol.2023.5033 PMC10666040

[6] Allison KH, Hammond MEH, Dowsett M et al (2020) Estrogen and Progesterone Receptor Testing in Breast Cancer: ASCO/CAP Guideline Update. J Clin Oncol 38(12):1346–1366. 10.1200/JCO.19.02309 31928404 10.1200/JCO.19.02309

[7] Jernström H, Rydén L (2024) Into the twilight zone – should ER-low breast cancer be treated as triple negative breast cancer? Lancet Reg Health - Europe 40:100896. 10.1016/J.LANEPE.2024.100896 38590941 10.1016/j.lanepe.2024.100896 PMC10999462

[8] Godoy-Ortiz A, Sanchez-Muñoz A, Parrado MRC et al (2019) Deciphering HER2 Breast Cancer Disease: Biological and Clinical Implications. Front Oncol 9(OCT):1124. 10.3389/FONC.2019.01124 31737566 10.3389/fonc.2019.01124 PMC6828840

[9] Qureshi Z, Altaf F, Jamil A, Siddique R, Fatima E (2024) Safety and Efficacy of Trastuzumab Deruxtecan for Metastatic HER2 + and HER2-low Breast Cancer. Am J Clin Oncol. Published online July 2. 10.1097/COC.0000000000001126 10.1097/COC.0000000000001126 38951994

[10] Modi S, Jacot W, Yamashita T et al (2022) Trastuzumab Deruxtecan in Previously Treated HER2-Low Advanced Breast Cancer. N Engl J Med 387(1):9–20. 10.1056/NEJMoa2203690 35665782 10.1056/NEJMoa2203690 PMC10561652

[11] Gaudio M, Jacobs F, Benvenuti C et al (2024) Unveiling the HER2-low phenomenon: exploring immunohistochemistry and gene expression to characterise HR-positive HER2-negative early breast cancer. Breast Cancer Res Treat 203(3):487–495. 10.1007/s10549-023-07151-3 37923964 10.1007/s10549-023-07151-3

[12] Geyer CE, Tang G, Mamounas EP et al (2018) 21-Gene assay as predictor of chemotherapy benefit in HER2-negative breast cancer. npj Breast Cancer 2018 4(1):1. 10.1038/s41523-018-0090-6 10.1038/s41523-018-0090-6 PMC6235896 30456299

[13] Sparano JA, Gray RJ, Makower DF et al (2020) Clinical Outcomes in Early Breast Cancer With a High 21-Gene Recurrence Score of 26 to 100 Assigned to Adjuvant Chemotherapy Plus Endocrine Therapy: A Secondary Analysis of the TAILORx Randomized Clinical Trial. JAMA Oncol 6(3):367–374. 10.1001/JAMAONCOL.2019.4794 31566680 10.1001/jamaoncol.2019.4794 PMC6777230

[14] Kalinsky K, Barlow WE, Gralow JR et al (2021) 21-Gene Assay to Inform Chemotherapy Benefit in Node-Positive Breast Cancer. N Engl J Med 385(25):2336–2347. 10.1056/NEJMOA2108873/SUPPL_FILE/NEJMOA2108873_DATA-SHARING.PDF 34914339 10.1056/NEJMoa2108873 PMC9096864

[15] Yao Zxiang, Lu Ljie, Wang Rjue et al (2014) Discordance and clinical significance of ER, PR, and HER2 status between primary breast cancer and synchronous axillary lymph node metastasis. Med Oncol 31(1):798. 10.1007/s12032-013-0798-y 24307349 10.1007/s12032-013-0798-y

[16] Kuncman W, Orzechowska M, Kuncman Ł, Kordek R, Taran K (2021) Intertumoral heterogeneity of primary breast tumors and synchronous axillary lymph node metastases reflected in IHC-assessed expression of routine and nonstandard biomarkers. Front Oncol 11. 10.3389/fonc.2021.660318 10.3389/fonc.2021.660318 PMC8595326 34804912

[17] Baroš IV, Tanasković N, Pellas U, Eri Ž, Tadić Latinović L, Tot T (2019) Internodal HER2 heterogeneity of axillary lymph node metastases in breast cancer patients. Bosn J Basic Med Sci. Published online April 2. 10.17305/bjbms.2019.3970 10.17305/bjbms.2019.3970 PMC6716091 30957723

[18] Janeva S, Parris TZ, Krabbe E et al (2023) Clinical relevance of biomarker discordance between primary breast cancers and synchronous axillary lymph node metastases. Clin Exp Metastasis 40(4):299. 10.1007/S10585-023-10214-W 37392277 10.1007/s10585-023-10214-w PMC10338601

[19] Shirman Y, Lubovsky S, Shai A (2023) HER2-Low Breast Cancer: Current Landscape and Future Prospects. Breast Cancer: Targets Therapy 15:605. 10.2147/BCTT.S366122 37600670 10.2147/BCTT.S366122 PMC10439285

[20] Shaaban AM, Kaur T, Provenzano E (2025) HER2-Low Breast Cancer—Current Knowledge and Future Directions. Med (B Aires) 61(4):644. 10.3390/MEDICINA61040644 10.3390/medicina61040644 PMC12028887 40282933

[21] Bardia A, Hu X, Dent R et al (2024) DESTINY-Breast06 Trial Investigators. Trastuzumab Deruxtecan after Endocrine Therapy in Metastatic Breast Cancer. N Engl J Med 391(22):2110–2122. 10.1056/NEJMoa2407086 39282896 10.1056/NEJMoa2407086

[22] Qureshi Z, Altaf F, Jamil A, Siddique R, Fatima E (2024) Safety and Efficacy of Trastuzumab Deruxtecan for Metastatic HER2 + and HER2-low Breast Cancer An Updated Systematic Review and Meta-Analysis of Clinical Trials. Am J Clin Oncology: Cancer Clin Trials 47(11):535–541. 10.1097/COC.0000000000001126 10.1097/COC.0000000000001126 38951994

[23] Gaudio M, Jacobs F, Benvenuti C et al (2023) Unveiling the HER2-low phenomenon: exploring immunohistochemistry and gene expression to characterise HR-positive HER2-negative early breast cancer. Breast Cancer Res Treat 203(3):487–495. 10.1007/S10549-023-07151-3 10.1007/s10549-023-07151-3 37923964

[24] Cherifi F, Cabel L, Bousrih C et al (2025) PROMENADE: pembrolizumab for early ER-low/HER2-negative breast cancer, real-world French cohort. ESMO Open 10(12):105907. 10.1016/J.ESMOOP.2025.105907 41297161 10.1016/j.esmoop.2025.105907 PMC12689199

[25] Steen S, Karlsson E, Björnheden I et al (2025) Pathological response to pembrolizumab-based neoadjuvant therapy in ER-low vs. ER-zero breast cancer: a Swedish population-based cohort study. Breast Cancer Res 27(1):213. 10.1186/S13058-025-02179-3/TABLES/2 41316480 10.1186/s13058-025-02179-3 PMC12670802

[26] Cardoso F, O’Shaughnessy J, Liu Z et al (2025) Pembrolizumab and chemotherapy in high-risk, early-stage, ER^+^/HER2^–^ breast cancer: a randomized phase 3 trial. Nat Med 31(2):442–448. 10.1038/s41591-024-03415-7 39838117 10.1038/s41591-024-03415-7 PMC11835712

[27] Nedergaard L, Haerslev T, Jacobsen GK (1995) Immunohistochemical study of estrogen receptors in primary breast carcinomas and their lymph node metastases including comparison of two monoclonal antibodies. APMIS 103(1):20–24. 10.1111/J.1699-0463.1995.TB01074.X 7695887 10.1111/j.1699-0463.1995.tb01074.x

[28] Aitken SJ, Thomas JS, Langdon SP, Harrison DJ, Faratian D (2009) Quantitative analysis of changes in ER, PR and HER2 expression in primary breast cancer and paired nodal metastases. Ann Oncol 21(6):1254–1261. 10.1093/annonc/mdp427 19858088 10.1093/annonc/mdp427

[29] Nedergaard L, Haerslev T, Jacobsen GK (1995) Immunohistochemical study of estrogen receptors in primary breast carcinomas and their lymph node metastases including comparison of two monoclonal antibodies. APMIS 103(1–6):20–24. 10.1111/j.1699-0463.1995.tb01074.x 7695887 10.1111/j.1699-0463.1995.tb01074.x

[30] Aitken SJ, Thomas JS, Langdon SP, Harrison DJ, Faratian D (2010) Quantitative analysis of changes in ER, PR and HER2 expression in primary breast cancer and paired nodal metastases. Ann Oncol 21(6):1254–1261. 10.1093/annonc/mdp427 19858088 10.1093/annonc/mdp427

[31] Yao ZX, Lu LJ, Wang RJ et al (2013) Discordance and clinical significance of ER, PR, and HER2 status between primary breast cancer and synchronous axillary lymph node metastasis. Med Oncol 31(1):798. 10.1007/S12032-013-0798-Y 10.1007/s12032-013-0798-y 24307349

[32] Kuncman W, Orzechowska M, Kuncman Ł, Kordek R, Taran K (2021) Intertumoral Heterogeneity of Primary Breast Tumors and Synchronous Axillary Lymph Node Metastases Reflected in IHC-Assessed Expression of Routine and Nonstandard Biomarkers. Front Oncol 11:660318. 10.3389/FONC.2021.660318/FULL 34804912 10.3389/fonc.2021.660318 PMC8595326

[33] Baroš IV, Tanasković N, Pellas U, Eri Ž, Latinović LT, Tot T (2019) Internodal HER2 heterogeneity of axillary lymph node metastases in breast cancer patients. Bosn J Basic Med Sci 19(3):242–248. 10.17305/BJBMS.2019.3970 30957723 10.17305/bjbms.2019.3970 PMC6716091

[34] Boolbol SK, Harshan M, Chadha M, et al (2019) Genomic comparison of paired primary breast carcinomas and lymph node macrometastases using the Oncotype DX Breast Recurrence Score^®^ test. 177:611–618. 10.1007/s10549-019-05346-1 10.1007/s10549-019-05346-1 PMC7472831 31302854

